# Lipoprotein(a): Role in Cardiovascular Risk and Advances in Novel Therapeutics

**DOI:** 10.7759/cureus.106817

**Published:** 2026-04-10

**Authors:** Adrián Murillo Sotela, Gloriana Orozco Loaiza, Paula Villalobos Villalobos, Jimena Alfaro Guerra

**Affiliations:** 1 General Medicine, Universidad de Costa Rica, San José, CRI

**Keywords:** antisense oligonucleotides, apolipoprotein(a), atherosclerosis, cardiovascular disease, lipoprotein(a), small interfering rna

## Abstract

Despite adequate primary and secondary prevention of cardiovascular events, significant residual risk remains. Part of this risk has been attributed to lipoprotein(a) (Lp{a}). This is a genetically determined lipoprotein that has been linked to cardiovascular disease. Its variability and pathogenicity are attributed to the unique apolipoprotein(a) (apo{a}) within the molecule. This protein has been related to proatherogenic, proinflammatory, prothrombotic, and procalcific mechanisms that favor cardiovascular disease (CVD). Although it is recognized as a causal factor in disease, there are currently no approved therapeutics targeting this lipoprotein. Current management focuses on aggressive control of traditional cardiovascular risk factors. Novel therapeutics targeting Lp(a), including small interfering ribonucleic acids (siRNAs) and antisense oligonucleotides (ASOs), showed promising results in phase 2 trials. Multiple therapeutics are currently undergoing phase 3 trials, promising to bring a solution to this unsolved issue.

## Introduction and background

Cardiovascular disease (CVD) is a term for a broad group of disorders affecting the heart and blood vessels [1]. A major component of CVD is atherosclerotic cardiovascular disease (ASCVD). ASCVD is driven mainly by atherosclerosis, a chronic inflammatory disease of the arterial wall [2,3]. This entity manifests mainly through acute events, like acute coronary syndromes and strokes [1]. As an epidemiological glance, CVD remains the leading cause of death globally. In 2022, an estimated 19.8 million people died from cardiovascular causes worldwide. This number represents 32% of all global deaths [1].

In the past couple of decades, cardiovascular mortality has significantly declined. This is mainly attributed to a stricter control of cardiovascular risk factors, such as smoking, and the widespread use of medical interventions, such as statins [3]. However, this decline has plateaued and even begun to reverse at alarming rates in recent years [4]. So, due to this stagnation and the fact that it remains the leading cause of death globally, CVD remains a latent issue to be solved.

Classically, CVD has been attributed to well-defined and recognized traditional risk factors that favor the development of ASCVD. Some of these factors include non-modifiable risk factors, such as age and sex, and modifiable risk factors, such as obesity and tobacco smoking [1,4-6].

Now, patients may still experience cardiovascular events despite adequate control of these traditional risk factors. This is where the term “residual cardiovascular risk” comes in. This refers to the latent risk of adverse cardiovascular events that persists in patients despite adequate primary and secondary prevention measures [7,8].

This residual cardiovascular risk is explained by non-conventional risk factors that contribute to the pathogenesis of ASCVD. These non-conventional factors are associated with pathways related to thrombosis, cholesterol metabolism, and inflammation. Of interest for this review is the genetically determined lipoprotein(a) (Lp{a}) [7,9].

Lp(a) was first described in 1963 as a variant of low-density lipoprotein (LDL) by Kåre Berg, a Norwegian geneticist. After its initial discovery, it took a decade to establish its association with CVD, and even longer to understand its true clinical significance [5,6,10].

Nowadays, through large-scale Mendelian randomization and genome-wide association studies (GWAS), Lp(a) has been recognized as a causal factor in CVD. More than 90% of Lp(a) levels are genetically determined. It has been proven that patients with genetic variants causing elevated levels of Lp(a) are more prone to CVD, especially ASCVD, calcific aortic valve stenosis (AVS), and even heart failure (HF). Conversely, carriers of variants associated with a loss-of-function of the lipoprotein(a) gene (LPA gene) display low levels of this lipoprotein and are protected against adverse cardiovascular events [5,7,11,12].

Globally, it is estimated that 20-30% of the world's population has clinically relevant elevated Lp(a) levels [7,13]. This statistic, in conjunction with the pathogenic role in CVD of this lipoprotein, highlights the relevance of incorporating the Lp(a) as a parameter to guide clinical decisions and the necessity to develop new therapeutic approaches to address this unsolved issue. The purpose of this narrative review was to analyze modern Lp(a) basic aspects, such as structure and pathogenesis, and clinical aspects, such as measurement and novel therapeutic targets.

## Review

Methods

For this narrative review, a comprehensive literature search was conducted, primarily using reputable scientific search engines, including PubMed, PubMed Central, Springer Nature Link, and ScienceDirect. The search focused on reviews, meta-analyses, and randomized controlled trials published between 2016 and 2026. The key search terms included as follows: Lp(a), apo(a), small interfering ribonucleic acids (siRNAs), antisense oligonucleotides (ASOs), and proprotein convertase subtilisin/kexin type 9 (PCSK9) inhibitors. Articles directly about lipoprotein(a) structure, pathophysiology, measurement, and treatment were prioritized. Only articles in the English language were selected. This narrative review aimed to synthesize the most relevant and current information regarding the described search.

Structure and genetics

At first glance, Lp(a) is highly similar to LDL. It is mainly synthesized in the liver and is composed of a lipidic core of cholesteryl esters and triacylglycerols, with an outer hydrophilic shell composed mainly of phospholipids, free cholesterol, and apolipoprotein B-100 (apoB-100) [6,7]. What makes Lp(a) unique, both in structural and biochemical properties, is the presence of apo(a), which is covalently linked to the apoB-100 particles [6,7,10,13].

This apolipoprotein is encoded by the LPA gene. This gene is located on the long arm of chromosome 6, specifically at the 6q21 region. It is inherited in an autosomal codominant pattern, and because of the gene's extreme genetic diversity, more than 80% of the global population is heterozygous. Because of this type of inheritance, neither allele masks the phenotypic expression of the other. Instead, both are expressed independently, and the final circulating Lp(a) concentration represents a sum of the synthetic output of both alleles [4,6,10,11].

Apo(a) is structurally homologous but not identical to plasminogen. It is mainly composed of loop-like structures called “kringle domains.” There are different types of these kringle domains, but apo(a) is composed of the 10 subtypes of kringle IV domains (KIV1 to KIV10) and a single kringle V domain (KV) [7,10,13].

**Figure 1:**
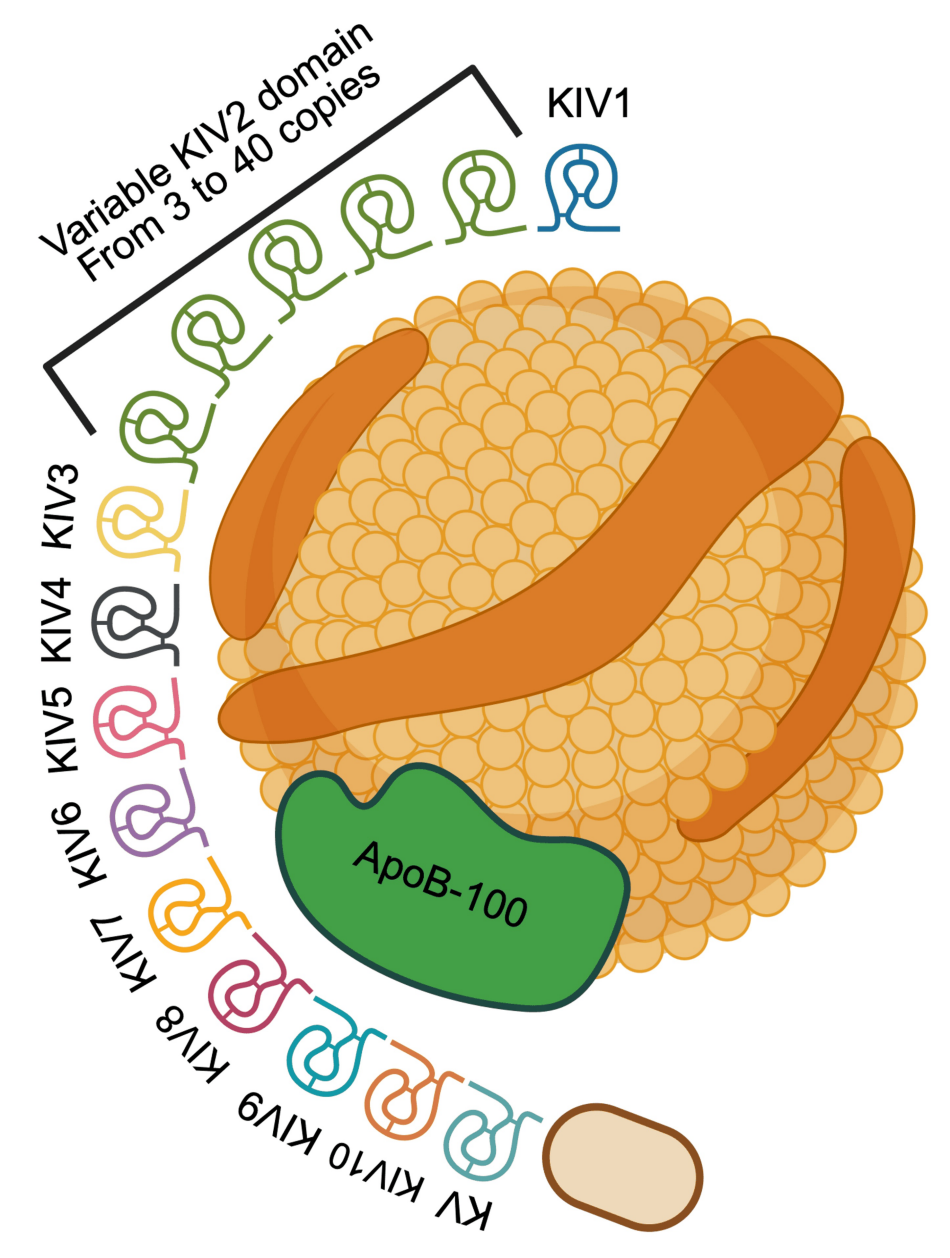
Apo(a) structure and kringle domains. The inner and outer composition of Lp(a) is highly similar to that of LDL. Within its outer shell, an Lp(a) molecule also has an apolipoprotein B-100 (apoB-100) molecule. Covalently linked to this apoB-100 is the apolipoprotein(a) (apo{a}), the distinctive characteristic of the Lp(a) molecule. It is analogous to plasminogen, composed mainly of loop-like structures called “kringle domains.” It comprises 10 subtypes of kringle IV domains (KIV1 to KIV10) and a single kringle V domain (KV). The variability of the apo(a) structure is due to the KIV2. This subtype of kringle IV domain is highly variable in the number of identical repeated copies, ranging from three to 40. This creates a vast range of Lp(a) isoforms, varying in weight [6,7,10,13]. This image is created by the author (Dr. Adrian Murillo Sotela) of this study, using BioRender pre-designed icons (BioRender.com/s7aathr).

These kringle domains are usually present as single copies in apo(a), with the exception of KIV2. This domain subtype is present in a highly variable number of identically repeated copies. The number of copies can range from three to 40 [6,10]. Due to this variation, Lp(a) can be present in over 40 different isoforms, each with a variable molecular weight ranging from 200 to 800 kDa [10,13]. An illustrative explanation of the apo(a) structure can be seen in Figure 1.

This variability in Lp(a) size is vital to understanding its plasma concentrations and clinical impact. There is a strong inverse correlation between Lp(a) size and its concentrations in plasma. This means that smaller isoforms, with fewer KIV2 copies, have higher plasma concentrations. On the other hand, larger isoforms, with higher numbers of KIV2 copies, have lower plasma concentrations [5,6,10]. The latter has been attributed to the fact that smaller isoforms of apo(a) have a more efficient handling in hepatocytes' endoplasmic reticulum, whereas larger isoforms have a prolonged handling in this organelle, causing difficult handling and making them more prone to intracellular degradation [5,10,14].

As previously mentioned, apo(a) is encoded by the LPA gene on chromosome 6. Within this gene, all kringle domains exist as single copies, except for KIV2. The sequence encoding this domain is present as a massive copy-number variation, making it highly variable and explaining the variation in apo(a) size within individuals. This variation in the number of KIV2 copies, and therefore in its size, explains 30-70% of the apo(a) plasma concentrations [5,10,14]. However, other genetic factors have an impact on apo(a) plasma concentrations. Individuals with the exact same isoform of Lp(a) may still have drastically different plasma concentrations, up to a 200-fold difference [5,10,14].

One explanation for the latter is the presence of single-nucleotide polymorphisms (SNPs) in the LPA gene [5,14]. Two of the most studied SNPs are rs10455872 and rs3798220, which are associated with very high Lp(a) concentrations. These ones do not alter the production or metabolism of Lp(a) directly; instead, they act as “tags” of small apo(a) isoforms and, therefore, higher Lp(a) concentrations [5]. Other SNPs located at splice sites of the KIV2 region, such as variants 4925G>A and 4733G>A, cause splicing defects or in-frame deletions that impair protein folding, causing instead reduced Lp(a) levels [5,14].

Beyond KIV2 copies and SNPs, other genetic factors, such as short tandem repeats, enhancer regions, and other genes (including APOE, APOH, and CETP), can alter Lp(a) plasma concentrations. In conjunction, up to 90% of Lp(a) plasma concentrations are explained by genetically determined factors [5,10,14].

Pathogenesis

Proinflammatory and Proatherogenic Effects of Lp(a)

Lp(a) is an important orchestrator of CVD, especially ASCVD. Its role as a causal marker of atherogenesis has already been established. Through different proatherogenic and proinflammatory mechanisms, it has been estimated that Lp(a) is five to six times more atherogenic than an LDL particle. This has been attributed mainly to the presence of apo(a), the distinct feature of Lp(a) [7].

Lp(a) particles are macromolecular complexes measuring approximately 250 angstroms in diameter, enabling them to cross through arterial walls to the subendothelial space, especially in cases of endothelium dysfunction [3,6,7]. Once in the subendothelial space, Lp(a) particles are retained to a greater extent than LDL. This is due to a high affinity between this particle and the extracellular matrix (ECM). This is mediated by a lysine binding site (LBS), located in the KIV10 domain of apo(a), that allows interactions with proteins and proteoglycans of the ECM [6,10].

The lipidic content of Lp(a) is also worth noting. It has been established that Lp(a) is the preferential carrier of oxidized phospholipids (OxPLs) in human circulation, carrying approximately 85% of all plasma OxPLs. Of these, around 50% are covalently bound to the apo(a), while the other 50% is carried in the lipidic core [10].

At the vascular level, OxPLs function as damage-associated molecular patterns (DAMPs) recognized by the innate immune system, promoting the initiation of sterile inflammation [6]. Once in the subendothelial space, OxPLs promote the expression of cellular adhesion molecules and the release of potent chemoattractants, thereby favoring the recruitment, adhesion, and migration of inflammatory cells. At the subendothelial level, macrophages also internalize OxPLs via scavenger receptors. This process favors the transformation of macrophages into foam cells. Further exposure of macrophages to OxPLs promotes the activation of caspase pathways, leading to further cytokine secretion and macrophage apoptosis. This cellular death favors the expansion of the necrosis core and plaque destabilization [6,7,10]. In summary, Lp(a) serves as a vehicle for OxPLs into the subendothelial spaces, thereby favoring proinflammatory and proatherogenic pathways that lead to further ASCVD [10].

Lp(a) also contributes to atherogenesis by favoring the recruitment of inflammatory cells. In vitro experiments have shown that it induces the expression of adhesion molecules in the endothelium, such as vascular cell adhesion molecule 1 (VCAM-1), E-selectin, and intercellular adhesion molecule 1 (ICAM-1) [6].

Chemotaxis is another factor regulated by Lp(a). It can directly induce chemotaxis, mainly through interactions mediated by the LBS. It also upregulates the production of inflammatory chemotactic molecules, including interleukin-1β (IL-1β), interleukin-8 (IL-8), tumor necrosis factor-𝛼 (TNF-𝛼), and monocyte chemotactic protein (MCP) [6].

**Figure 2:**
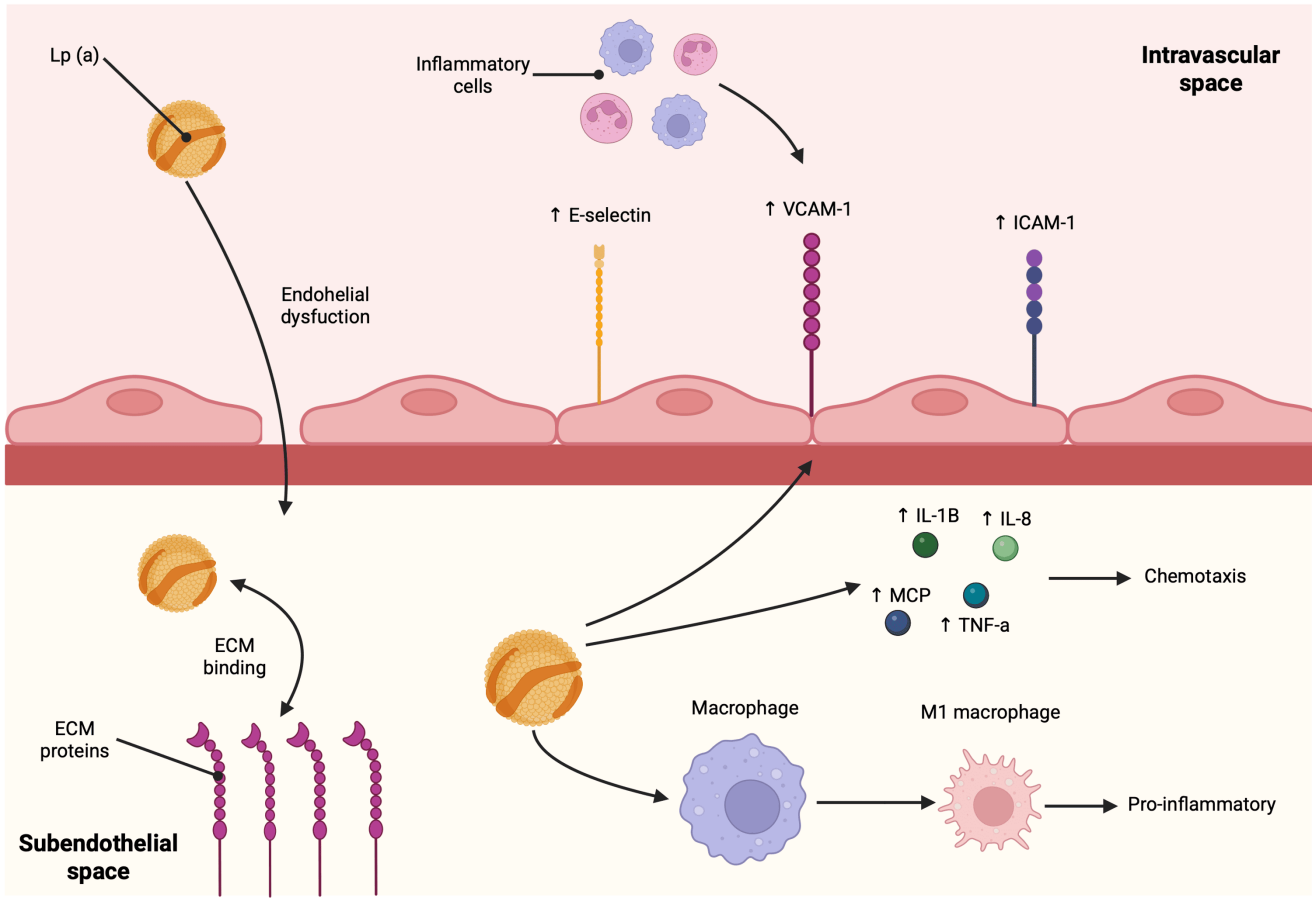
Proinflammatory and proatherogenic effects of Lp(a). Apo(a) is mainly responsible for Lp(a) proinflammatory and proatherogenic effects. Through its LBS in KIV10, apo(a) is able to interact and bind to extracellular matrix (ECM) proteins and proteoglycans, allowing a greater retention in the subendothelial space. Lp(a) and its contents, mainly OxPLs, promote different inflammatory and atherogenic mechanisms. It favors the expression of adhesion molecules in the endothelium, such as E-selectin, vascular cell adhesion molecule 1 (VCAM1), and intercellular adhesion molecule 1 (ICAM-1). It also promotes the secretion of inflammatory markers that prompt further chemotaxis, such as interleukin-1β (IL-1β), interleukin-8 (IL-8), tumor necrosis factor-α (TNF-α), and monocyte chemotactic protein (MCP). Finally, it also induces changes in cell phenotypes, favoring more proinflammatory phenotypes [6,7,10]. This image is created by the author (Dr. Adrian Murillo Sotela) of this study, using BioRender pre-designed icons (BioRender.com/2qh12p6).

Lastly, Lp(a) also favors the expression of more proinflammatory phenotypes in different tissues and cells. For example, the exposure of monocytes to Lp(a) induces the expression of proinflammatory (M1-type) macrophage phenotype, which is characterized by improved adhesive capabilities and augmented secretion of chemoattractants, stimulating further inflammation [6]. The previous mechanisms are schematized in Figure 2.

Thrombotic and Antifibrinolytic Mechanisms Associated With Lp(a)

In vitro studies have shown that Lp(a) is an important regulator of hemostasis pathways, such as fibrinolysis and thrombosis. However, in vivo studies have shown that it is not a causal factor of venous thromboembolism and that Lp(a) lowering failed to diminish clot lysis time. Nevertheless, there are potential roles and mechanisms through which Lp(a) promotes thrombus formation in the vasculature. Its prothrombotic and antifibrinolytic properties seem to act mainly at sites of atherothrombosis secondary to ASCVD rather than being a generalized hypercoagulability factor [10,11,15].

Regarding fibrinolysis, the apo(a) component of Lp(a) is structurally similar to plasminogen, including its LBS. Due to this homology, it causes a competition for the fibrin-binding site. This reduces the normal binding of plasminogen and tissue plasminogen activator (t-PA), reducing the formation of plasmin and, thus, the degradation of fibrin [6,10,15]. Also, under normal circumstances, plasminogen and t-PA bind to fibrin, creating a ternary complex that catalyzes the production of plasmin. Lp(a) can bind to this structure, creating a quaternary complex that is far less effective at catalyzing the conversion to plasmin [6,15].

In addition, Lp(a) can also cause a diminution in plasmin production in indirect ways. It has been shown that Lp(a) is capable of inducing augmented expression of plasminogen activator inhibitor-1 (PAI-1) in nearby endothelial cells, prompting further antifibrinolytic mechanisms [6,10].

Beyond hindering clot breakdown, Lp(a) is also characterized by its procoagulant properties in the vasculature. It has been shown that, through its apo(a), it is capable of inducing platelet activation and aggregation. This has been attributed to the ability of apo(a) to interact with platelet receptors, like protease-activated receptor 1 (PAR-1) and the glycoprotein IIb/IIIa (GPIIb/IIIa). The interaction with this receptor theoretically hinders collagen and ADP-induced platelet aggregation, but the net effect is procoagulant [6,7,10,15].

**Figure 3:**
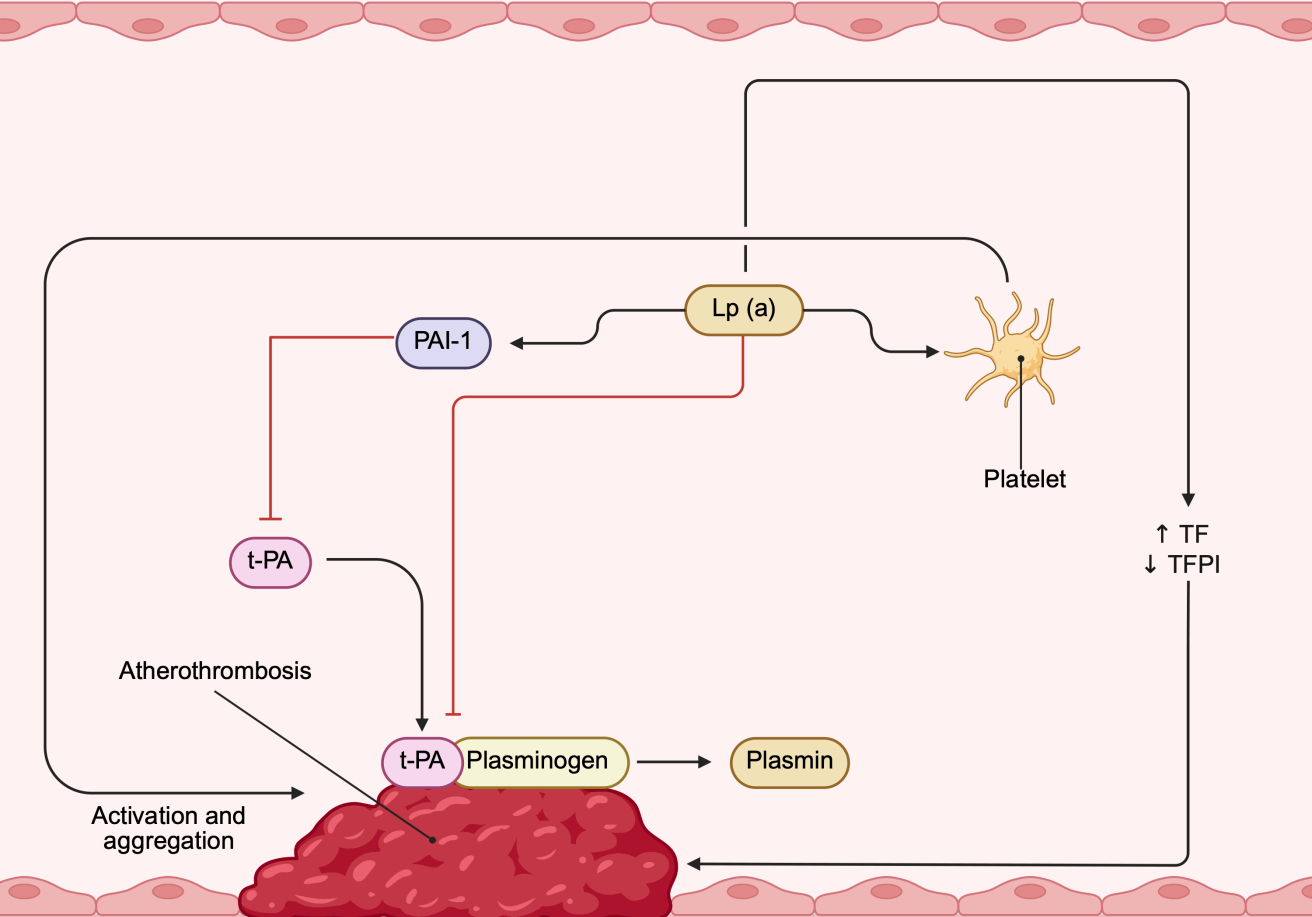
Thrombotic and antifibrinolytic mechanisms associated with Lp(a). Lp(a) prothrombotic and antifibrinolytic mechanisms are seen mainly at atherothrombosis sites. Normally, tissue plasminogen activator (t-PA), plasminogen, and fibrin form a ternary complex that efficiently converts plasminogen to plasmin. Lp(a) can bind to these elements to create a quaternary complex that is far less effective in said conversion. Lp(a) has effects at the endothelial level, where it can stimulate antifibrinolytic molecules, like plasminogen activator inhibitor-1 (PAI-1), inhibiting the action of t-PA. It has been shown that apo(a) can interact with platelet PAR-1 and GPIIB/IIIA, thereby favoring platelet activation and aggregation. Finally, Lp(a) can induce endothelial and inflammatory cells to augment the expression of tissue factor (TF), a primary initiator of coagulation, and tissue factor pathway inhibitor (TFPI), an endothelium-derived anticoagulant [6,7,10,15]. This image is created by the author (Dr. Adrian Murillo Sotela) of this study, using BioRender pre-designed icons (BioRender.com/b6hnkct).

Lp(a) has also been shown to have effects on secondary hemostasis. In vitro studies have shown that Lp(a) and OxPLs augment the expression in monocytes and macrophages of tissue factor (TF), a main primary initiator of the coagulation cascade [6,15,16]. It can also inactivate endothelium-derived anticoagulants, like tissue factor pathway inhibitor (TFPI). Lp(a), especially through the apo(a) LBS, can directly bind to TFPI, neutralizing the molecule and its anticoagulant properties [6,10,15]. The previous mechanisms are schematized in Figure 3.

Lp(a) as a Key Mediator of Aortic Valve Calcification

Several studies have confirmed the role of Lp(a) as an independent causal risk factor for calcific aortic valve stenosis (AVS). Initially, the pathophysiology is the same as atherosclerosis, in which Lp(a) infiltrates damaged valvular endothelium and delivers a high payload of OxPLs, prompting a proinflammatory and procalcific reaction [10,11,13,15-17]. OxPLs are metabolized by phospholipase A2 (PLA2) to create lysophosphatidylcholine (LysoPC). Posteriorly, LysoPC is metabolized by the autotaxin (ATX), an enzyme carried by Lp(a), to form lysophosphatidic acid (LysoPA) [10,17].

**Figure 4:**
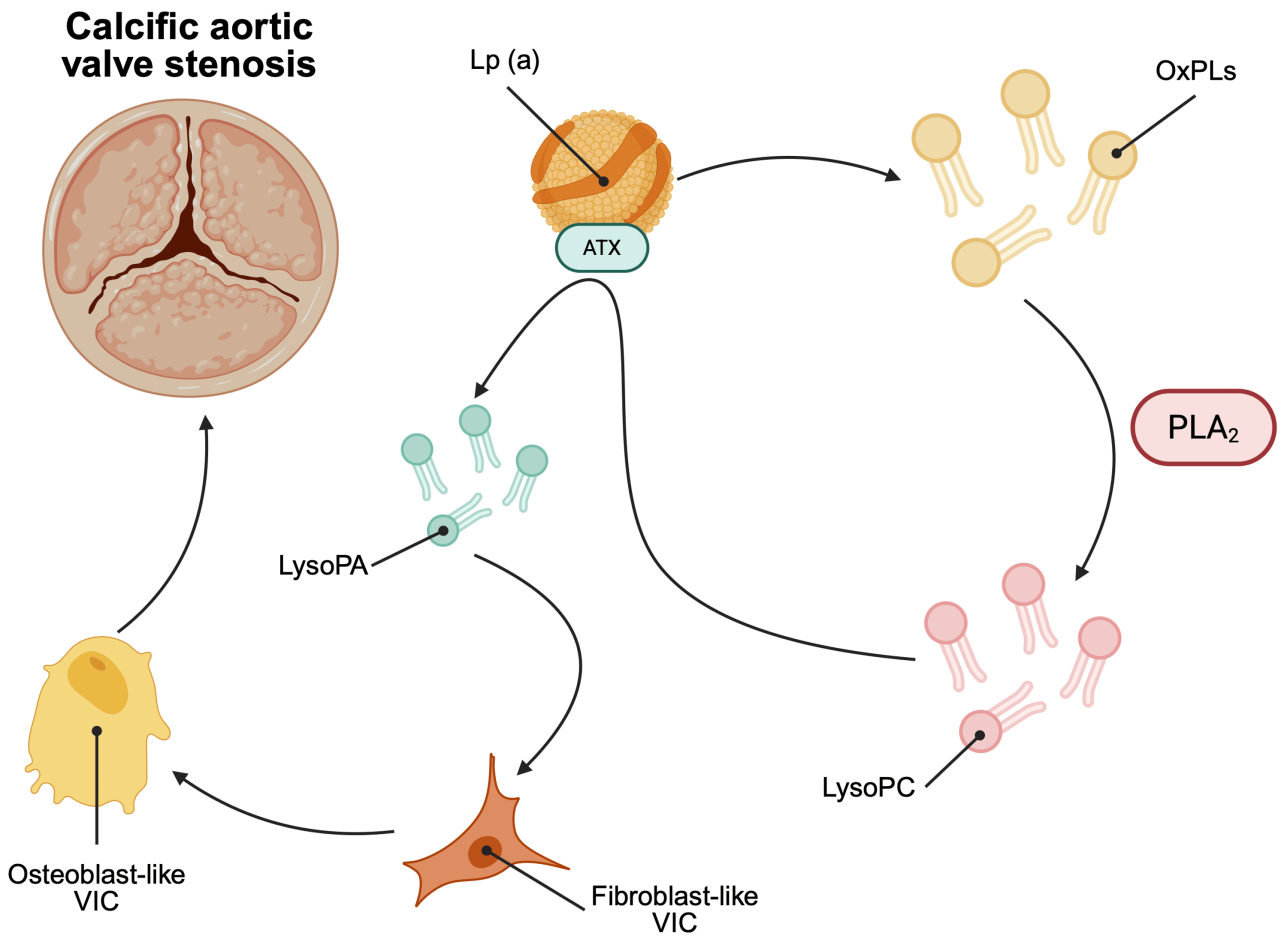
Lp(a) as a key mediator of aortic valve calcification. Lp(a) is the main carrier of oxidized phospholipids (OxPLs). These are metabolized by the phospholipase A2 (PLA2), creating lysophosphatidylcholine (LysoPC). This is later metabolized by the autotaxin (ATX) enzyme into lysophosphatidic acid (LysoPA), a highly bioactive molecule that favors osteoblast-like phenotype of valvular interstitial cells (VIC), leading to calcific aortic valve stenosis [10,11,13,15-17]. This image is created by the author (Dr. Adrian Murillo Sotela) of this study, using BioRender pre-designed icons (BioRender.com/dy6adur).

The LysoPA is a highly bioactive molecule that binds to different receptors on valve endothelial cells and valve interstitial cells (VIC), stimulating different pathways that cause inflammation and calcification. Worth noting, the action of LysoPA in VIC triggers a phenotype switch, from fibroblast-like cells to osteoblast-like cells. This change in cell programming favors the expression of genes and proteins that upregulate the deposition of calcific material in the valve, leading to rapid micro- and macrocalcification of the aortic valve [10,11,17]. The previous mechanism is schematized in Figure 4.

Heart Failure: An Emerging Association With Lp(a)

Since its discovery, Lp(a) has been investigated for its association with numerous cardiovascular diseases. Heart failure is not an exception. Singh et al. conducted a systematic review and meta-analysis of Mendelian randomization studies in which they found that Lp(a) was linked to a higher risk of HF, with an odds ratio of 1.064 (95% CI: 1.043-1.085; p<0.001) [12]. Nomura et al. conducted a study to analyze the overall incidence of HF by ethnicity using data from three separate cohorts [18]. They found that the incidence of HF, both HF with preserved ejection fraction (HFpEF) and HF with reduced ejection fraction (HFrEF), among white participants increased in accordance with Lp(a) levels, reporting a hazard ratio of 1.19 for Lp(a) ≥50 mg/dL (95% CI: 1.07-1.34; p=0.002). This relation was not observed for Black participants, in which they reported a hazard ratio of 0.93 for Lp(a) ≥50 mg/dL (95% CI: 0.78-1.11; p=0.415). Importantly, the association was no longer statistically significant after excluding cases of myocardial infarction, highlighting the relevance of ASCVD in this association [18]. Finally, Maliha et al. conducted a retrospective study to assess the association between Lp(a) and different HF subtypes. They found that patients with Lp(a) >75 nmol/L had a statistically significantly higher prevalence of HFpEF (p=0.004), without differences between ethnicities. The association was not seen for HF with mid-range ejection fraction (HFmrEF) nor HFrEF [19].

As previously established, Lp(a) is strongly associated with ASCVD. The previously mentioned studies agree that the association between Lp(a) and HF is mainly mediated by coronary artery disease, a well-established risk factor for HF. ASCVD, through myocardial scarring and remodeling, favors the development of different subtypes of HF. Other factors related to Lp(a), such as aortic valve stenosis, arterial vascular stiffness, and proinflammatory properties, have been linked to the development of HF [12,18,19].

Explanations for the differences observed across races and ethnicities remain unclear. A first hypothesis for these differences is the genetic heterogeneity of the LPA gene. Another potential explanation is differences in cardiovascular risk factors for HF across races. For example, Black individuals have higher rates of hypertension, obesity, and diabetes. These can take the lead role over the Lp(a) mediated effects, diminishing the association in this ethnic group [12,18,19].

In summary, Lp(a) is associated with an increased risk of developing HF. Differences between races are evident in studies, with a more prominent effect in white individuals. Further studies that clarify the exact mechanisms by which Lp(a) contributes to HF and confirm differences across ethnicities are necessary.

Measuring Lp(a)

The majority of measurements of Lp(a) in studies are performed through immunoassays, such as enzyme-linked immunoabsorbent assays (ELISA). The measurement poses a challenge, mainly due to its structural heterogeneity, the lack of universal calibrators, and the lack of standard reference material [7,11,20].

A noteworthy limitation is the type of antibodies used in measuring immunoassays. Ideally, immunoassays should use isoform-independent monoclonal antibodies that target a non-repetitive component of the apo(a), such as the KIV9. This is with the purpose that each Lp(a) molecule is recognized only once, independently of its size [4,7,11].

However, this type of antibody is hard and costly to obtain. Therefore, many commercial assays use polyclonal antibodies that bind to KIV2 domains, which vary in size and repeat number. The latter causes larger Lp(a) particles to bind more antibodies, and smaller ones bind fewer antibodies, causing overestimation and underestimation of Lp(a) levels, respectively [7,11].

Another key point of measurement is the unit in which the test is reported. The first option is the measurement of total mass per given volume of blood, specifically mg/dL. The second option is the measurement of actual Lp(a) particles per a given volume of blood, specifically nmol/L [4,21].

Currently, guidelines recommend using molar units to study Lp(a). This accounts for possible molecular weight differences among individuals due to their variable KIV2 repeats and thus Lp(a) size. However, measurement in mg/dL is most widely available in laboratories. Its use is considered acceptable for clinical purposes [4,7,13].

An important aspect to highlight is that conversion between mg/dL and nmol/L is strongly discouraged, because there is no reliable universal conversion factor that takes into account the size variation of the Lp(a) molecules [11,21].

Indications of who to test have varied over time and issuing entities. According to the latest clinical guidelines, including the 2025 Focused Update of the 2019 European Society of Cardiology (ESC)/European Atherosclerosis Society (EAS) Guidelines for the Management of Dyslipidemias and the 2026 American College of Cardiology (ACC)/American Heart Association (AHA) Guideline on the Management of Dyslipidemia, universal Lp(a) screening is recommended at least once in every adult lifetime. According to these sources, the main goal is to identify patients with highly elevated Lp(a) levels (e.g., >180 mg/dL or >430 nmol/L), because these patients carry a risk of ASCVD equal to or higher than the risk seen in heterozygous familial hypercholesterolemia (HeFH) [3,4,7,11,17,22].

If a more targeted screening is preferred, Lp(a) measurement is strongly recommended in the following circumstances: personal history of premature ASCVD, recurrent ASCVD events despite optimal therapy (high residual risk), familial hypercholesterolemia (FH) or other genetic forms of dyslipidemia, family history of premature ASCVD or elevated Lp(a) levels, and history of calcific AVS. Additionally, Lp(a) can be measured in patients classified as having a borderline or intermediate CVD risk in 10-year ASCVD scales. In this context, Lp(a) acts as a risk enhancer that can reclassify patients' CVD risk, guiding clinical and therapeutic decisions [6,7,11,22].

As established before, up to 90% of Lp(a) circulating levels are genetically determined, with little variation due to environmental or lifestyle factors. Therefore, current recommendations state that serial measurements of Lp(a) are not necessary and that a one-time only quantification is sufficient. However, there are a few exceptions to the latter, in which re-testing is indicated. A first indication is menopause, as Lp(a) may increase after its onset. A second condition is initial Lp(a) borderline levels, also referred to as the “grey zone” (30-50 mg/dL or 75-125 nmol/L), in which re-testing can reclassify into a lower or higher risk category. A third indication is patients with conditions that can alter Lp(a) metabolism, such as kidney or hepatic diseases [4,6,7,11,22].

Even though Lp(a) is recognized as an ASCVD causal factor, it is important to clarify that the overall risk also depends on the total global CVD risk, taking into account lifestyle and traditional CVD risk factors. For example, a patient with high Lp(a) levels but low CVD absolute risk does not have the same overall risk as a patient with high Lp(a) levels and high CVD absolute risk [5].

For clinical stratification purposes, thresholds had to be established to categorize patients according to their Lp(a) levels. The “low risk zone” (<30 mg/dL or <75 nmol/L) is considered normal and is not associated with increased CVD risk. The “grey zone” (30-50 mg/dL or 75-125 nmol/L) is where CVD risk begins to rise; therapeutic decisions must also take into account other factors, such as absolute risk and traditional risk factors. The “high risk zone” (50 ≥mg/dL or 125 ≥nmol/L) is already associated with a high CVD burden; approximately 20-30% of the global population falls into this category. The “very high risk zone” (≥180 mg/dL or 430 ≥nmol/L) is considered as extremely high Lp(a) levels and carries a severe lifetime risk of CVD, comparable to those with HeFH [3,4,5,11,17,22].

Management

Intensification of Baseline Prevention

As mentioned earlier, Lp(a) levels are predominantly determined by genetics. This means that lifestyle has minimal to no impact on concentrations. Also, as of today, there are currently no approved pharmacological therapies to lower Lp(a) levels. Taking this into account, the mainstream modern management focuses on aggressively controlling the global CVD risk and managing existing risk factors [4,5,11,13,23].

As specified in the latest American and European guidelines, Lp(a) levels ≥50 mg/dL or ≥125 nmol/L are considered a risk-enhancing factor. This increases the 10-year ASCVD risk and guides decision-making regarding management. Also, guidelines state that optimal control of modifiable risk factors is imperative to reduce overall ASCVD risk in patients with Lp(a) above these levels [4,22].

The previous approach rests on the premise that the excess CVD risk conferred by the genetically determined Lp(a) can be mitigated by rigorous control of other CVD risk factors, especially LDL. This includes appropriate screening, statin initiation in those who require it, and setting strict, controlled lipid targets (e.g., LDL <55 mg/dL in high-risk patients) [3,4,11].

This baseline prevention must also transcend beyond lipid management. Other factors associated with inflammation and endothelial dysfunction must be controlled. These include, but are not limited to, strict glycemic control in diabetic patients, rigorous blood pressure control in case of hypertension, and absolute tobacco smoking suspension [4,11].

The Paradoxical Effect of Statins on Lp(a) Levels

Statins are a mainstream treatment for dyslipidemias. They act by inhibiting hydroxymethylglutaryl-coenzyme A (HMG-CoA), the main rate-limiting enzyme in cholesterol synthesis. By downregulating cholesterol synthesis in hepatocytes, it bolsters the expression of LDL receptors on the cell surface. This favors the hepatic uptake of LDL and apoB-containing lipoproteins [3].

Initially, a positive association between Lp(a) and the LDL receptor was established. This was mainly supported by the presence of the apoB protein in the Lp(a) structure and in vitro observations that identified a high affinity between them. However, extensive data, including meta-analyses, have demonstrated that statin therapy does not lower Lp(a) levels. Instead, a paradoxical relationship has been observed, with Lp(a) plasma concentrations increasing by 10-20% with statin therapy [6,9,10,17,24].

This increase in levels has been explained mainly by in vitro experiments showing increased hepatic production of Lp(a). Hepatocyte exposure to statins seems to produce a time and dose-dependent increase in transcription of Lp(a), depicted by an increased expression of LPA messenger ribonucleic acid (mRNA), and therefore, apo(a) [9,16].

Even though statins show this paradoxical increase in Lp(a) levels, they should not be suspended in the management of patients. As mentioned before, the mainstream management of elevated Lp(a) levels, due to a lack of approved targeted treatments, focuses on reducing global CVD risk. Therefore, a primary therapeutic goal is to achieve profound LDL reductions with high-intensity statins, which have been shown to reduce major adverse cardiovascular events (MACE) in these vulnerable patients. In conclusion, the use of statins should not be suspended in these patients, because the benefits achieved outweigh any theoretical harm associated with the Lp(a) increase [6,11].

Ezetimibe as a Neutral Agent in Lp(a) Management

Ezetimibe is a non-statin lipid-lowering agent, mainly used to decrease circulating LDL. This medication inhibits the Niemann-Pick C1-like 1 (NPC1L1) cholesterol transfer protein in the small intestine. A decrease in dietary cholesterol leads to cholesterol depletion in hepatocytes, resulting in upregulation of LDL receptors, which capture and diminish circulating LDL [6,16,20].

Overall, several meta-analyses have concluded that ezetimibe has little to no effect on Lp(a) levels. Unlike statin therapy, ezetimibe use has not been linked to an upregulation of Lp(a). Thus, it is considered a neutral medication [12,20,23]. Therefore, its role is restricted to controlling the global CVD risk in these patients. According to guidelines, ezetimibe is indicated in cases in which LDL treatment goals cannot be achieved using statins only. As monotherapy, it can achieve LDL reductions of 15-22%, while when co-administered with statins, it can achieve an additional reduction of 21-27% [3,20,22,24].

PCSK9 Inhibitors: Modest Reduction of Lp(a) of Uncertain Significance

PCSK9 is a critical regulator of cholesterol homeostasis. Circulating PCSK9 binds to the LDL receptor, triggering internalization and lysosomal degradation of the receptor. By doing this, it reduces the hepatocytes' capacity to clear LDL from circulation [3,7]. 

PCSK9 inhibitors are monoclonal antibodies that bind selectively to circulating PCSK9 proteins, neutralizing them and preventing their binding to LDL receptors, or siRNAs that interrupt the synthesis of the protein itself. Blunting this interaction upregulates the number of LDL receptors available and thus enhances LDL and apo-B clearance [3,7].

Because of its similarity with statins that upregulate LDL receptors, it was thought that PCSK9 inhibitors would not have an impact on Lp(a) levels. However, these molecules have shown potential reductions of Lp(a) levels of 15-30% [7,11,16,23].

The mechanism through which PCSK9 inhibitors accomplish this reduction remains unclear. Different hypotheses have been postulated. The first one states that the profound upregulation of LDL receptors and the very low levels of LDL achieved by PCSK9 inhibitors force LDL receptors to clear Lp(a) molecules. A second one, based on in vitro studies, suggests that inhibition of PCSK9 may have a direct impact on hepatic synthesis of apo(a) and, thereby, Lp(a). Finally, the third hypothesis establishes that PCSK9 inhibitors cause a profound clearance of apoB-100 particles, causing a reduction in its bioavailability and hindering apo(a) production [6,7,10,11,16,23].

Evolocumab is a monoclonal antibody directed against PCSK9. Its effects on Lp(a) were mainly studied in the subanalysis of the "The Further Cardiovascular Outcomes Research with PCSK9 Inhibition in Subjects With Elevated Risk" (FOURIER) trial. It resulted in an average reduction of 26.9% of Lp(a) levels after 48 weeks and an absolute cardiovascular risk reduction of 2.41% in patients with Lp(a) levels >50 mg/dL. Alirocumab is another monoclonal antibody against PCSK9. Its effects on Lp(a) were mainly studied in the subanalysis of the "Evaluation of Cardiovascular Outcomes After an Acute Coronary Syndrome During Treatment With Alirocumab" (ODYSSEY-OUTCOMES) trial. The results were similar to those in the FOURIER study, with an average reduction of 23-29% of Lp(a) levels and an 3.7% absolute cardiovascular risk reduction in patients with Lp(a) levels >60 mg/dL. Lastly, inclisiran is the siRNA of the PCSK9 inhibitors. Its effects were analyzed in the ORION trials, where it achieved Lp(a) reductions between 15% and 25% depending on the dose [7,11].

Even though these studies showed reductions of Lp(a) levels and a reduction of absolute cardiovascular risk in patients, they were designed to analyze the effects of LDL lowering. Meaning that the benefits observed cannot be directly attributed to the Lp(a) lowering. Besides, these trials included mostly patients with low Lp(a) levels, potentially diluting the possible effect of PCSK9 inhibitors in Lp(a) [7,11].

Considering the above, PCSK9 inhibitors are not specifically approved by regulatory agencies as a primary medication to lower Lp(a). Therefore, the use of this class of medications should be restrained as indicated in guidelines - very-high risk patients, including those with FH or progressive ASCVD, who have high Lp(a) and fail to achieve LDL target levels despite maximally tolerated statin therapy [3,4].

Apheresis: The Extracorporeal Removal of Lp(a)

Lipoprotein apheresis is an extracorporeal therapeutic modality that is being used in the management of progressive ASCVD due to elevated Lp(a). Most available systems clear all apoB-containing particles, including LDL. Other, more specialized systems are capable of selectively filtering Lp(a) [7,25].

A single apheresis session can achieve Lp(a) lowerings as high as 60-80% post-procedure. Because of the steady hepatic synthesis of the lipoprotein, weekly or biweekly sessions are necessary to achieve mean reductions of 25-50% of circulating Lp(a) [23,25].

Due to the high cost, invasive nature of the procedure, and some ethical considerations, large-scale cardiovascular outcome trials regarding apheresis are scarce. However, there are some data supporting possible benefits of this therapeutic approach [23,25]. A sham-controlled double-blind trial in 2017 demonstrated that weekly apheresis over three months improved myocardial perfusion reserve and exercise capacity in 20 subjects with refractory angina and Lp(a) >50 mg/dL. Also, there are registry data showing that the CVD events are lower following the initiation of regular apheresis. However, these data are limited due to the lack of a control group [25].

The indications for apheresis are highly regulated and vary by geographic region. As established by regulatory agencies, an isolated elevated Lp(a) is not an indication for initiating apheresis. Instead, there is consensus that it should be reserved as a last resort in patients with elevated Lp(a) with progressive CVD despite optimal management of other risk factors. Specifically, the Federal Drug Administration (FDA) approved apheresis for patients with Lp(a) ≥60 mg/dL if they also have FH and coronary artery disease or peripheral artery disease [4,11,23,25].

In conclusion, apheresis seems to be an effective therapy, but because of its high costs, logistics, and impact on patients' quality of life, it currently serves as a bridge and rescue therapy for high-risk patients until novel medications directed against Lp(a) are available for management [4,7,25].

siRNA: A Next-Generation Therapy for Lp(a)

siRNAs are a novel and potent therapeutic class that have been developed for the management of Lp(a). These molecules consist of double-stranded, non-coding RNA sequences. To ensure hepatic specificity and avoid systemic toxicity, these molecules are covalently bound to N-acetylgalactosamine (GalNAc), thereby favoring selective endocytosis by hepatocytes via the asialoglycoprotein receptor (ASGPR-1) [7,17,23].

Once inside the cell, the double-stranded RNA dissociates. The active antisense strand binds to an intracellular protein known as the ribonucleic acid-induced silencing complex (RISC). This complex acts continuously, targeting, cleaving, and degrading mRNA encoding apo(a). By diminishing the synthesis of apo(a), consequently, the assembly of Lp(a) is lowered. It is known that the RISC complex is highly stable, providing extended pharmacological effect and allowing infrequent dosing (e.g., every three to six months) [7,13,17,23].

Olpasiran is the most clinically advanced siRNA currently under development. Its pharmacodynamic effects and safety were studied in the "Olpasiran Trials of Cardiovascular Events and Lipoprotein(a) Reduction - Dose Finding Study" (OCEAN(a)-DOSE) trial. This was a multicenter, randomized, double-blind, phase 2 trial. It compared different subcutaneous doses of olpasiran every 12 or 24 weeks against placebo in 281 patients with ASCVD and Lp(a) levels greater than 150 nmol/L [7,23,26].

At the 36-week follow-up, it was shown that olpasiran caused deep dose-dependent reductions in circulating Lp(a) - 70.5% with a 10 mg dose every 12 weeks, 97.4% with a 75 mg dose every 12 weeks, 101.1% with a 225 mg dose every 12 weeks, and 100.5% with a 225 mg dose every 24 weeks. It was also reported that LDL and apoB showed some reduction with olpasiran treatment [7,23,26].

Currently, the "Olpasiran Trials of Cardiovascular Events and Lipoprotein(a) Reduction - Outcomes Study" (OCEAN(a)-Outcomes) trial (NCT05581303) is ongoing to test the hypothesis that massive reductions of Lp(a) with olpasiran translate into a reduction of the residual atherothrombotic risk. It is a double-blind, multicenter, and placebo-controlled trial. It enrolled a total of 7,300 patients with a history of ASCVD and Lp(a) levels greater than 200 nmol/L. The primary endpoint is major adverse cardiovascular events (MACE). The study began in December 2022, and it's expected to conclude in March 2028 [7,10,17,27].

Lepodisiran is another miRNA currently being tested. "A Study of Lepodisiran in Participants With Elevated Lipoprotein(a)" (ALPACA) was a multicenter, randomized, and placebo-controlled phase two trial. A total of 320 patients older than 40 years with Lp(a) concentrations greater than 175 nmol/L were randomized to receive different doses of lepodisiran or placebo at baseline and on day 180. The primary endpoint was the time-averaged percent change of Lp(a) from day 60 to 180. The results for Lp(a) lowering were as follows: 40.8% for a 16 mg dose, 75.2% for a 96 mg dose, and 93.9% for a 400 mg dose [28].

The "A Cardiovascular Outcomes Study to Evaluate the Effect of Lepodisiran on Major Adverse Cardiovascular Events in Patients With Elevated Lipoprotein(a)" (ACCLAIM-Lp{a}) trial (NCT06292013) is a phase 3 trial that started in March 2024. It is a multicenter, double-blind, placebo-controlled trial to study the MACE reduction with lepodisiran. It enrolled a total of 17.300 participants older than 18 years with established ASCVD or older than 55 years at very high risk of first CVD, all of them with Lp(a) levels greater than 175 nmol/L. This criterion of inclusion makes the ACCLAIM-Lp(a) trial the first to study Lp(a) lowering in primary prevention. It is currently ongoing and is expected to conclude in March 2029 [7,10,16,29].

A third medication in this category is Zerlasiran. In the "Assessment of Lipoprotein(a) lowering In Cardiovascular Disease With SLN360" (ALPACAR-360) second-phase trial, a total of 180 patients with established ASCVD were randomized to receive different Zerlasiran doses at different time intervals. The primary endpoint was time-averaged percent change in Lp(a) concentration from baseline to week 36. For each group, the reductions were as follows: 85.6% for 450 mg every 24 weeks, 82.8% for 300 mg every 16 weeks, and 81.3% for 300 mg every 24 weeks [23,30]. As of today, there is no active phase 3 study for Zerlasiran.

ASOs: Gene Silencing of Lp(a)

ASOs are a novel pharmacological class highly targeted to reduce Lp(a) levels. These molecules consist of synthetic, single-stranded analogs of nucleic acids, typically between 13 and 20 nucleotides in length. As with siRNAs, ASO molecules are covalently bound to a GalNAc, granting ASOs great affinity to the ASGPR-1 and, therefore, hepatic uptake specificity [7,10,13,23].

Following the hepatic endocytosis of ASOs, these molecules translocate to the nucleus. Here, they bind to their complementary target: apo(a) mRNA. Together, they form a double-strand ASO-mRNA complex that is recognized by ribonuclease H1 (RNase H1), an endogenous intracellular endonuclease. This enzyme cleaves the sense strand (the mRNA strand), preventing the synthesis of apo(a). Moreover, the ASO strand remains intact after cleavage, allowing it to restart the described process [7,10,13,23].

Pelacarsen is the most clinically advanced ASO. In "Apolipoprotein(a)-LRx, an Antisense Oligonucleotide Targeting Lipoprotein(a), in Patients With Cardiovascular Disease" (AKCEA-APO(a)-LRx) second phase trial, a total of 286 patients with established ASCVD and Lp(a) greater than 150 nmol/L received various doses of pelacarsen at different time intervals and were compared against placebo. The main endpoint was the percentage change in Lp(a) from baseline to six months. The reductions reported in Lp(a) were as follows: 35% for the 20 mg every four weeks group, 56% for the 40 mg every four weeks group, 58% for the 20 mg every two weeks group, 72% for the 60 mg every four weeks group, and 80% for the 20 mg every week group. Additionally, it also showed modest reductions in LDL, apoB, and OxPLs levels [7,10,17,23,31].

Currently, pelacarsen is being evaluated in the phase 3 trial "A Study of Pelacarsen (TQJ230) in Participants With Established Cardiovascular Disease" (Lp{a} HORIZON) (NCT04023552). It is a randomized double-blind, placebo-controlled, multicenter trial evaluating the impact of pelacarsen in MACE. It includes 8323 patients with established ASCVD and Lp(a) greater than 70 mg/dL. It is expected to finish soon in June 2026 [32]. Pelacarsen is also going to be tested in "A Study of Pelacarsen (TQJ230) in Participants With Calcific Aortic Valve Stenosis" (Lp{a} FRONTIERS CAVS) (NCT05646381). This phase 3 trial aims to determine if pelacarsen can halt disease progression in patients with mild to moderate calcific AVS. Currently, this trial is undergoing recruitment, and it is estimated to conclude in March 2030 (Table 1) [16,33].

**Table 1:** Comparison of novel therapeutic drugs targeting Lp(a). Main characteristics and study status of the main novel drugs directly targeting Lp(a). siRNA: small interfering ribonucleic acid; ASO: antisense oligonucleotide; RISC: RNA-induced silencing complex, RNase H1: ribonuclease H1; Lp(a): lipoprotein(a) This table was created by Dr. Adrian Murillo Sotela based on the references [26-32].

Characteristic	Olpasiran	Lepodisiran	Zerlasiran	Pelacarsen
Drug class	siRNA	siRNA	siRNA	ASO
Molecular target	Apo(a) mRNA	Apo(a) mRNA	Apo(a) mRNA	Apo(a) mRNA
Mechanism of action	siRNA-RISC mediated mRNA degradation	siRNA-RISC mediated mRNA degradation	siRNA-RISC mediated mRNA degradation	RNase H1-mediated ASO-mRNA degradation
Route of administration	Subcutaneous	Subcutaneous	Subcutaneous	Subcutaneous
Dosing (second phase)	10 mg every 12 weeks, 75 mg every 12 weeks, 225 mg every 12 weeks, and 225 every 24 weeks	16 mg every 180 days, 96 mg every 180 days, and 400 mg every 180 days	300 mg every 16 weeks, 300 mg every 24 weeks, and 450 mg every 24 weeks	20 mg every 4 weeks, 40 mg every 4 weeks, 60 mg every 4 weeks, 20 mg every 2 weeks, and 20 mg every week
Mean percentage Lp(a) lowering (second phase)	66.9% to 96.9%	40.8% to 93.9%	81.3% to 85.6%	35.0% to 80.0%
Status	Phase 3 study ongoing	Phase 3 study ongoing	No active phase 3 ongoing	Phase 3 study ongoing
Phase 3 study	OCEAN(a) - outcomes trial (NCT05581303)	ACCLAIM-Lp(a) trial (NCT06292013)	-	Lp(a) HORIZON (NCT04023552)
Due date	March 2028	March 2029	-	June 2026

Gene Editing Approaches Targeting Lp(a) 

The Clustered Regularly Interspaced Short Palindromic Repeats (CRISPR) system, combined with the CRISPR-associated protein 9 (Cas9), is a complex gene-editing technology that aims to eradicate genetically based diseases, such as Lp(a) disease. The Cas9 is a nuclease that induces a directed double-strand break in the DNA, in this case of the LPA gene. After this, the cell attempts to repair the break using an error-prone mechanism known as non-homologous end joining (NHEJ). This repair process causes insertions or deletions in the gene sequence, disrupting the gene's reading frame and transcription [5,7,34].

The most clinically advanced CRISPR-Cas9 agent being studied for lowering is CTX320. Preclinical in vivo studies in animals showed reductions of up to 90% of circulating Lp(a) levels with favorable tolerability and a safety profile [7,16,31,34]. However, despite its therapeutic potential, the CRISPR-Cas9 technology still faces many challenges. Some of these include the following: off-target mutagenesis, irreversibility of the procedure, accessibility to the technology, and ethical dilemmas [34].

## Conclusions

Lp(a) has been recognized as a causal factor in CVD, contributing to the residual risk that continues to trouble modern management. A sound understanding and continuous updates on the basic aspects, significance, and management of Lp(a) are imperative for today's clinical practice. Even though there are currently no targeted therapies, its quantification in patients can help guide management and encourage physicians to intensify therapeutic efforts in order to counter the residual risk attributable to this genetically determined lipoprotein. Currently, trials of novel therapeutics targeting Lp(a) are ongoing, promising to address the knowledge gap in Lp(a) treatment.
